# Supplementary material for: Trends in immobilization following fracture surgery of the wrist and hand in Sweden: A 16-Year Analysis from 2008 to 2023
Source: PLoS One. 2025 Jun 25;20(6):e0324367. doi: 10.1371/journal.pone.0324367 (PMC12193958; doi:10.1371/journal.pone.0324367)
Supplement: Attachment — Submitted filename: Response.docx [file pone.0324367.s001.docx]

Response

1. Please complete all items on the Human Participants Research Checklist that are relevant for your submission, by following this link:  <https://eur01.safelinks.protection.outlook.com/?url=http%3A%2F%2Fjournals.plos.org%2Fplosone%2Fs%2Ffile%3Fid%3Ddc11%2FPLOSOne_Human_Subjects_Research_Checklist.docx&data=05%7C02%7Cviktor.schmidt%40ki.se%7C71bec733fcef4f78406308dd45f5488b%7Cbff7eef1cf4b4f32be3da1dda043c05d%7C0%7C0%7C638743642106788325%7CUnknown%7CTWFpbGZsb3d8eyJFbXB0eU1hcGkiOnRydWUsIlYiOiIwLjAuMDAwMCIsIlAiOiJXaW4zMiIsIkFOIjoiTWFpbCIsIldUIjoyfQ%3D%3D%7C0%7C%7C%7C&sdata=dbHlqZFmSTIdE1DodVZJ4SAXrTt%2BjOG%2B7ATmdoO04Gk%3D&reserved=0> (Contact us at plosone@plos.org if you cannot access the document.) There may be overlap between the checklist items and other queries listed below; please address any duplicated queries both in your response email and on the checklist itself. Upload the completed Human Participants Research Checklist as file type “Other” when you re-submit your manuscript. This document is for internal journal use only and will not be published if your article is accepted. The requested information will help us to assess whether your submission complies with PLOS ONE’s policies and adheres to applicable reporting standards. Note that your manuscript may be rejected if you provide incomplete or inadequate responses to the checklist questions and that changing the
   ‘Section/Category’ of your article does not affect this requirement.

We have uploaded the document.

2. For studies reporting research involving human participants, PLOS ONE requires authors to confirm that this specific study was reviewed and approved by an institutional review board (ethics committee) before the study began. Please provide the specific name of the ethics committee/IRB that approved your study, or explain why you did not seek approval in this case. Once you have amended this/these statement(s) in the Methods section of the manuscript, please add the same text to the “Ethics Statement” field of the submission form (via “Edit Submission”).

It ways in the Methods section ”Ethics: The study was performed using open-source data and was therefore not subject to ethical review.”

## Moreover, the data used in this study is obtained from the website of the SNBHW and is publicly available for anyone to download and use.

3. Please provide additional details regarding participant consent. In the ethics statement in the Methods and online submission information, please ensure that you have specified (1) whether consent was informed and (2) what type you obtained (for instance, written or verbal, and if verbal, how it was documented and witnessed). If your study included minors, state whether you obtained consent from parents or guardians. If the need for consent was waived by the ethics committee, please include this information.

The data used in this study is obtained from the website of the SNBHW and is publicly available for anyone to download and use. The study was performed using open-source data and was therefore not subject to ethical review or consent.
