# Supplementary material for: Trends in immobilization following fracture surgery of the wrist and hand in Sweden: A 16-Year Analysis from 2008 to 2023
Source: PLoS One. 2025 Jun 25;20(6):e0324367. doi: 10.1371/journal.pone.0324367 (PMC12193958; doi:10.1371/journal.pone.0324367)
Supplement: Attachment — Submitted filename: Point-by-point response to reviewers.docx [file pone.0324367.s002.docx]

**Manuscript**

**Response to Reviewers**

Dear Editor,

The authors thank you for considering the present manuscript. We appreciate the effort that the reviewers dedicated to improving the manuscript and are grateful for the insightful comments on and valuable improvements to the paper.
We have addressed the reviewers’ remarks below and the corresponding changes have been made in the revised manuscript.

The authors hope that you will find the manuscript suitable for future publication in PLOS ONE.

Best regards,

Viktor Schmidt
Corresponding author

Editor

1. Please ensure that your manuscript meets PLOS ONE's style requirements, including those for file naming. The PLOS ONE style templates can be found at

<https://journals.plos.org/plosone/s/file?id=wjVg/PLOSOne_formatting_sample_main_body.pdf> and

[https://journals.plos.org/plosone/s/file?id=ba62/PLOSOne_formatting_sample_title_authors_affiliations.pdf](https://eur01.safelinks.protection.outlook.com/?url=https%3A%2F%2Ftrack.editorialmanager.com%2FCL0%2Fhttps%3A%252F%252Fjournals.plos.org%252Fplosone%252Fs%252Ffile%253Fid%3Dba62%252FPLOSOne_formatting_sample_title_authors_affiliations.pdf%2F1%2F010f01963c18f1d5-c05bb104-154d-44ca-8448-c5f7666c1ca4-000000%2F9C2HmKMldrmPaBJZn1LEHg9HjQt4-4nyg2qhdFuZhhc%3D207&data=05%7C02%7Cviktor.schmidt%40ki.se%7C7fb3055c836349b9df5508dd7c8186dc%7Cbff7eef1cf4b4f32be3da1dda043c05d%7C0%7C0%7C638803618240953732%7CUnknown%7CTWFpbGZsb3d8eyJFbXB0eU1hcGkiOnRydWUsIlYiOiIwLjAuMDAwMCIsIlAiOiJXaW4zMiIsIkFOIjoiTWFpbCIsIldUIjoyfQ%3D%3D%7C0%7C%7C%7C&sdata=K3N8YBrvta6XSKFARVx33wjS1DDM3ER5aDqEIP%2BoC9I%3D&reserved=0).

**Author response:** Thank you. We have edited accordingly throughout the manuscript.

2. Thank you for uploading your study's underlying data set. Unfortunately, the repository you have noted in your Data Availability statement does not qualify as an acceptable data repository according to PLOS's standards.

At this time, please upload the minimal data set necessary to replicate your study's findings to a stable, public repository (such as figshare or Dryad) and provide us with the relevant URLs, DOIs, or accession numbers that may be used to access these data. For a list of recommended repositories and additional information on PLOS standards for data deposition, please see <https://journals.plos.org/plosone/s/recommended-repositories>.

**Author response:** Thank you. We have uploaded the data to: <https://figshare.com/s/1ffe20f7f9a839b5f4c9>

DOI: 10.6084/m9.figshare.28842941

3. Please review your reference list to ensure that it is complete and correct. If you have cited papers that have been retracted, please include the rationale for doing so in the manuscript text, or remove these references and replace them with relevant current references. Any changes to the reference list should be mentioned in the rebuttal letter that accompanies your revised manuscript. If you need to cite a retracted article, indicate the article’s retracted status in the References list and also include a citation and full reference for the retraction notice.

**Author response:** We have reviewed the reference list. Removed a duplicate (9 and 31).

**Reviewer #1:**

*1. Expand the discussion on potential drivers of regional differences (e.g., surgeon training, resource availability) beyond speculative explanations.
Acknowledge that the lack of fracture severity/surgical technique data limits causal interpretations of immobilization trends.*

**Author response:** Thank you for your review and good input. We have rewritten the discussion and added discussion on potential drivers. However, we cannot go beyond speculative explanations as we cannot know for sure what the reasons are since we lack granular data and this is a retrospective study.

**L201 added:** Regional differences in immobilization practices may stem in part from variability in surgeon training and practice settings. Surgeons who trained at high-volume academic centers or completed specialized hand fellowships might adopt more progressive, evidence-based postoperative protocols, including earlier mobilization. By contrast, those practicing in smaller community or rural hospitals—often with more generalized training—may adhere to traditional conservative approaches (e.g. routine casting for several weeks). Indeed, the lack of universally endorsed protocols means postoperative care often reflects the surgeon’s personal training and preferences (25). A recent review highlighted that “where an orthopedic surgeon trained, whether they obtained a hand or upper extremity fellowship, and their current practice location are all potential influencing factors” in immobilization strategy (25). Thus, if one Swedish region’s orthopedic staff has a strong academic or subspecialty background, they may be more comfortable with limited immobilization and early motion.

**L277 changed to:** The study has several limitations. While the Swedish NPR provides comprehensive data, potential inaccuracies in coding and reporting may affect results. The results of this study could in part be explained by a change in registration of cast fixation after surgical treatment of these fractures. The study does not account for fracture severity, specific fracture types, detailed fracture location, surgical techniques or patient comorbidities, all of which can influence immobilization decisions. These are critical confounders that limit causal inference about why immobilization trends differed by region. For example, a region treating a higher proportion of complex or unstable fractures (e.g. comminuted intra-articular injuries) would appropriately use longer immobilization on average, independent of surgeon preference.

Moreover, regional differences in resources and clinical practices are inferred rather than directly measured. Our findings are consistent with that understanding, but the inability to adjust for severity or technique means we cannot definitively claim that training, infrastructure, or culture caused the immobilization differences – only that they are plausible contributing factors. This limitation highlights a need for more detailed, prospective data to confirm the reasons behind regional practice patterns.
